# A refined radiological classification of anterior clinoid process pneumatization

**DOI:** 10.3389/fsurg.2026.1726314

**Published:** 2026-04-13

**Authors:** Wilairat Kankuan Kaewborisutsakul, Anukoon Kaewborisutsakul, Chin Taweesomboonyat, Nuttha Sanghan

**Affiliations:** 1 Division of Health and Applied Sciences, Faculty of Science, Prince of Songkla University, Songkhla, Thailand; 2 Neurological Surgery Unit, Division of Surgery, Faculty of Medicine, Prince of Songkla University, Songkhla, Thailand; 3 Division of Radiology, Faculty of Medicine, Prince of Songkla University, Songkhla, Thailand

**Keywords:** anterior clinoid process, computed tomography, endoscopic endonasal approach, optic canal decompression, optic strut, planum sphenoidale, radiological classification, skull base

## Abstract

**Introduction:**

The anterior clinoid process (ACP) is a critical anatomical landmark during skull base surgery. However, ACP pneumatization poses several risks during anterior clinoidectomy, including cerebrospinal fluid (CSF) leakage and optic nerve injury. Existing classification systems inadequately address clinically significant variations such as those involving the optic strut or planum sphenoidale. Therefore, this study aimed to determine the prevalence and morphological patterns of ACP pneumatization in a Thai population and propose a refined radiological classification system based on the route and extent of pneumatization.

**Methods:**

A retrospective computed tomography (CT)-based study was conducted on 400 ACPs from 200 patients aged ≥10 years. Pneumatization patterns were categorized into eight subtypes based on the pneumatization route (optic strut, planum sphenoidale, or both) and the degree of ACP involvement (≤50% or >50%). ACP morphometric data and associated bone variations were also assessed.

**Results:**

ACP pneumatization was observed in 30.8% of ACPs, with bilateral involvement in 5% of cases. The most frequent subtype was isolated optic strut pneumatization (subtype 1, 16%), followed by limited ACP involvement via the optic strut (subtype 2a, 6%). Planum-based and combined subtypes (3a and 4b) were uncommon (<4%). Male patients demonstrated significantly greater ACP base width (9.09 ± 1.61 mm vs. 8.54 ± 1.39 mm; *p* = 0.015) and length (13.23 ± 1.72 mm vs. 12.61 ± 1.64 mm; *p* = 0.010) than females. Middle clinoid processes and interclinoid calcifications were present in 5.8% and 8.8% of patients, respectively.

**Conclusion:**

ACP pneumatization, particularly via the optic strut, is a common anatomical variation. The proposed eight-subtype classification provides a nuanced framework for preoperative imaging description and communication. Although prior classifications were largely discussed in the context of transcranial approaches, the observed pneumatization patterns may also be relevant to endoscopic endonasal anatomy, particularly regarding optic canal exposure and potential sinonasal communication. Prospective surgical correlation studies are warranted to determine concordance with intraoperative findings and to clarify clinical relevance.

## 1 Introduction

The anterior clinoid process (ACP) is a small but surgically significant projection of the lesser sphenoid wing that forms a part of the roof of the optic canal and the lateral wall of the cavernous sinus. It is closely related to critical neurovascular structures, including the optic nerve; ophthalmic artery; clinoid and supraclinoid segments of the internal carotid artery; and cranial nerves III, IV, V1, and VI along the lateral wall or within the cavernous sinus (1, 2). Owing to these intimate relationships, ACP removal during anterior clinoidectomy is an essential step in the management of paraclinoid aneurysms, clinoidal or tuberculum sellae meningiomas, and optic canal decompression (3, 4). However, the procedure remains technically demanding, and anatomical variations such as ACP pneumatization may predispose patients to complications, including cerebrospinal fluid (CSF) leakage and optic nerve injury (5, 6).

From a developmental perspective, the ACP is formed by the ossification of the sphenoid bone, whereas pneumatization follows the maturation of the sphenoid sinus. This process does not occur before age 10 and is closely linked to the growth of the sphenoid sinus thereafter (3, 7). The pneumatization route most often extends from the sphenoid sinus, but in some cases, it proceeds from the ethmoid sinus via the optic strut or planum sphenoidale sinus (6). These variations may render the ACP thin-walled, increasing intraoperative injury risk; however, in certain situations, they provide an additional surgical corridor, facilitating decompression of the optic canal or exposure of the distal dural ring (8).

ACP pneumatization prevalence varies widely among populations and studies, ranging from 4% to nearly 40% (3, 5, 6, 9, 10). Several classification systems have been proposed for describing these patterns. Abuzayed et al. introduced a degree-based system ranging from absent to complete pneumatization (5), whereas Mikami et al. emphasized the route of pneumatization through the optic strut, planum sphenoidale, or both (6). More recently, da Costa et al. combined route and extent into a comprehensive classification system that recognized optic strut pneumatization as a distinct and clinically important subtype (3). Although these systems have advanced the field, to our knowledge, none have captured all possible combinations of pneumatization, and their reproducibility across populations remains limited (11).

Most importantly, existing data are derived almost entirely from Japanese, Turkish, or Brazilian cohorts, and ethnic variability in skull base anatomy has been demonstrated in related structures, such as the caroticoclinoid and interclinoid ligaments (12, 13). To our knowledge, there has been no systematic evaluation of ACP pneumatization in Thai patients, leaving a knowledge gap that may affect preoperative risk assessment in this population.

Therefore, this study was designed to evaluate the prevalence and distribution of ACP pneumatization in Thai patients aged ≥10 years. Based on our findings, we also propose a refined eight-subtype classification that incorporates both the pneumatization route and degree of ACP involvement. This system aims to provide a more comprehensive radiological framework for the preoperative evaluation of both transcranial and endoscopic endonasal approaches.

## 2 Methods

### 2.1 Study design and population

This retrospective cross-sectional radiological study was conducted in the Neurological Surgery Unit of a Thai University Hospital. Consecutive Thai patients aged ≥10 years who underwent cranial computed tomography (CT) between January 2016 and December 2024 were screened. Of these, 200 were included using sex-stratified sampling to achieve equal numbers of males (*n* = 100) and females (*n* = 100). The exclusion criteria were as follows: (1) craniofacial trauma or fracture; (2) intracranial pathology distorting the parasellar region; (3) prior cranial surgery; and (4) congenital or acquired skull deformity. Each ACP was analyzed separately with two observations per patient.

### 2.2 Sample size estimation

The minimum sample size was estimated based on the reported ACP pneumatization prevalence of 24% by Düz et al. (10). At a 95% confidence level and 5% margin of error, at least 281 ACPs (141 patients) were required. This study exceeded this threshold and included 200 patients (400 ACPs).

### 2.3 CT acquisition and image review

All CT scans were performed at our institution using a Toshiba Aquilion Prime 160-slice scanner. Acquisition parameters included a collimation of 40 × 0.5 mm, pitch factor of 0.625, matrix size of 512, and field of view of 200–220 mm. The tube voltage was 120 kVp, with a current of 320–400 mA. Images were reconstructed at 0.5–1.0 mm thickness and reformatted in axial, coronal, and sagittal planes, with three-dimensional reconstructions generated when required. ACP pneumatization was defined as the presence of paranasal sinus air cells within the ACP, as identified by areas of air-equivalent attenuation.

### 2.4 Morphometric measurements

**Figure 1:**
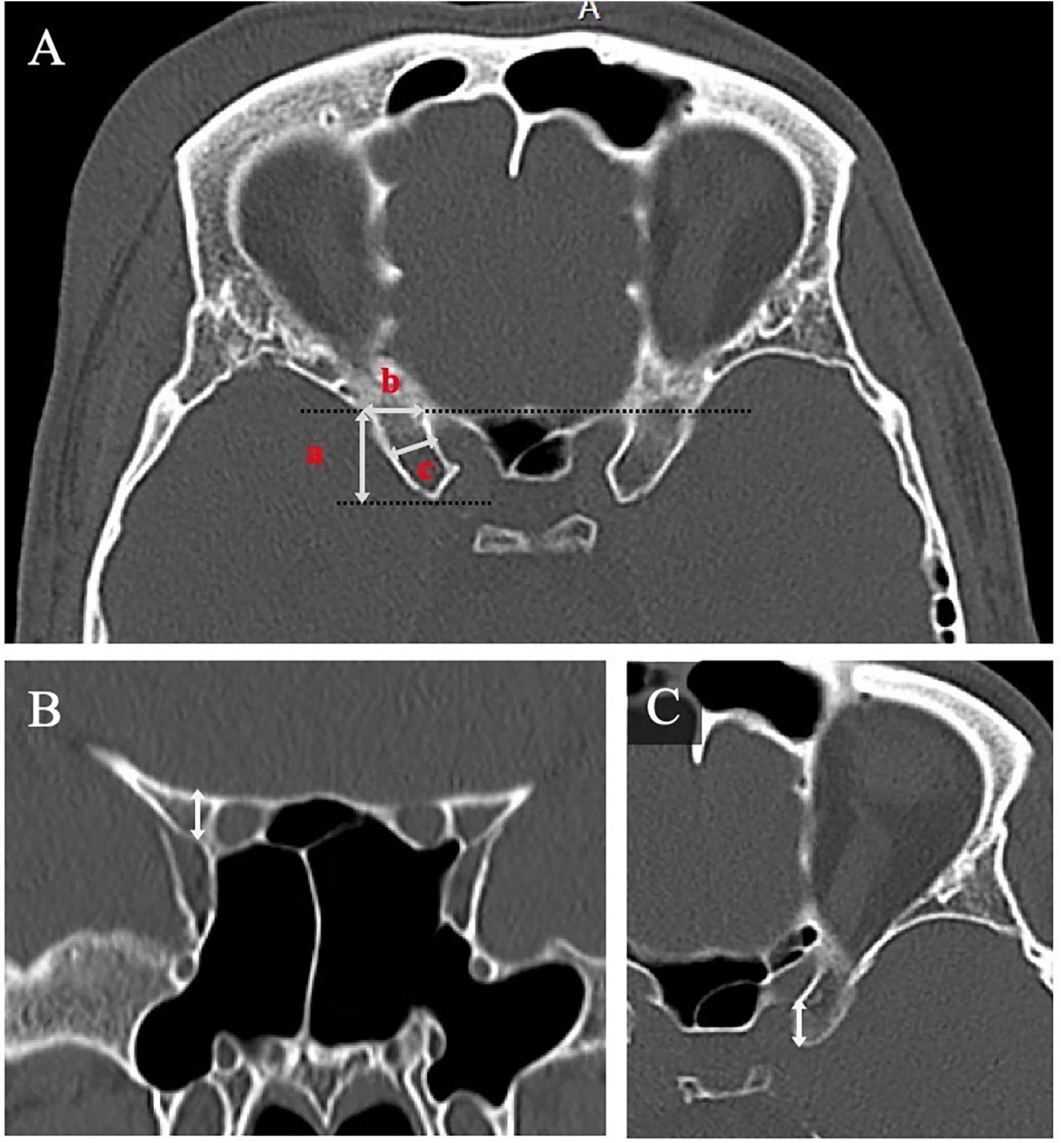
Morphometric measurements of the anterior clinoid process (ACP) and examples of Type 0 and Type 1 pneumatization **(A)** axial CT image demonstrating right ACP length **(a)**, base width **(b)**, and midpoint width **(c) (B)** coronal CT image demonstrating ACP base thickness (arrow). The right ACP shows no pneumatization (Type 0), while the left ACP demonstrates pneumatization limited to the optic strut (Type 1). **(C)** axial CT image demonstrating ACP tip-to-optic strut distance (arrow).

ACP dimensions were measured on high-resolution CT images using standardized anatomical landmarks (Figure 1). Parameters included: ACP length (a, Figure 1A), distance from the ACP baseline to the tip, and ACP base width (b, Figure 1A), distance from the medial optic canal to the lateral insertion on the sphenoid wing; midpoint width (c, Figure 1A), maximal width at the midpoint of the ACP; Base thickness (arrow, Figure 1B), thickness of the ACP at the base on the coronal plane lateral to the optic canal; ACP–optic strut distance (arrow, Figure 1C).

### 2.5 Classification of pneumatization

**Figure 2:**
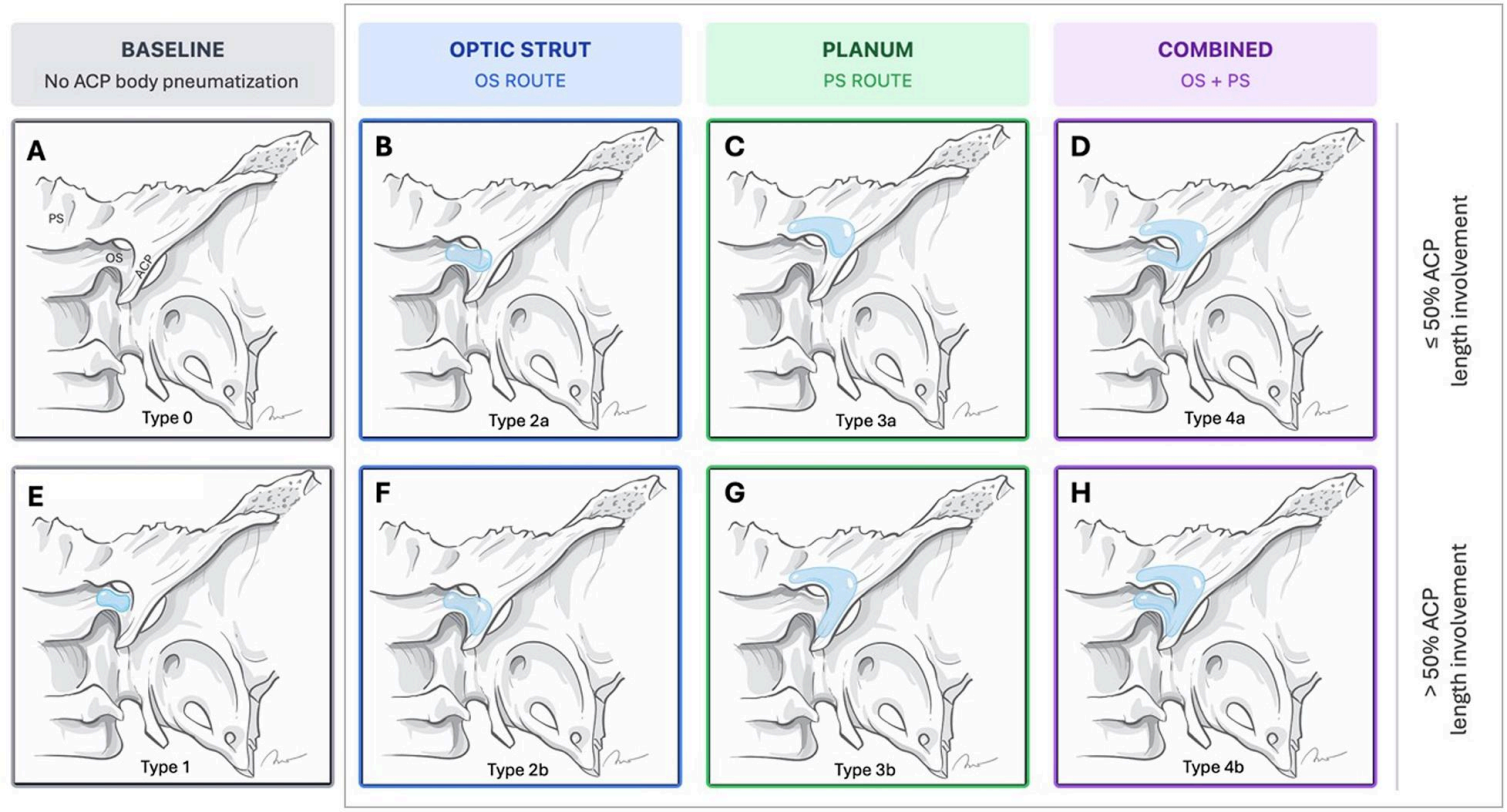
The refined classification of anterior clinoid process (ACP) pneumatization. The classification is structured as a logical matrix defined by the route of pneumatization (columns) and the extent of ACP length involvement (rows). **(A,E)** Type 0 **(A)** represents complete absence of pneumatization, and Type 1 **(E)** represents pneumatization limited to the optic strut (OS) without ACP body involvement. **(B,F)** Pneumatization through OS route: Type 2a **(B)** shows ≤50% ACP involvement, and Type 2b **(F)** shows >50% involvement. **(C,G)** Pneumatization through planum sphenoidale (PS) route: Type 3a **(C)** shows ≤50% ACP involvement, and Type 3b **(G)** shows >50% involvement. **(D,H)** Pneumatization through combined routes (OS + PS): Type 4a **(D)** shows ≤50% ACP involvement, and Type 4b **(H)** shows >50% involvement. All images are bone-window computed tomography (CT) scans.

Pneumatization was first categorized according to previously established systems, including (6) (route-based) (5), (volume-based), and (3) (combined route and degree). To address limitations of these systems, we applied a refined eight-subtype classification (Figure 2, Table 1,) that distinguishes pneumatization route (optic strut, planum sphenoidale, or combined) and stratifies the extent of ACP involvement using a ≤ 50% vs. >50% threshold.

**Table 1:** The refined classification of anterior clinoid process (ACP) pneumatization.

Type	Route and extent of ACP Pneumatization
0	Absence of any pneumatization
1	Pneumatization is limited to the optic strut without involving the ACP
2a	Pneumatization via the optic strut with ≤50% of ACP length involvement
2b	Pneumatization via the optic strut with >50% of ACP length involvement
3a	Pneumatization through the planum sphenoidale with ≤50% ACP length involvement
3b	Pneumatization through the planum sphenoidale with >50% ACP length involvement
4a	Pneumatization via both planum and optic strut with ≤50% ACP length involvement
4b	Pneumatization via both planum and optic strut with >50% ACP length involvement

The extent threshold was defined relative to ACP length. On multiplanar reformatted CT (axial, coronal, and sagittal planes), reviewers identified the entry route of aeration and assessed the longitudinal extent of aerated sinus cells extending from that route toward the ACP tip. Pneumatization was classified as ≤50% when aeration involved half or less of the ACP length and as >50% when aeration extended to more than half of the ACP length.

### 2.6 Data collection and management

All CT scans were evaluated by two independent reviewers (AK and WK). Morphometric measurements used the mean of both reviewers' values, and differences >1 mm were jointly re-evaluated to reach consensus. Categorical classifications (ACP subtype, middle clinoid process, interclinoid calcification) required total agreement, with discrepancies resolved by a third investigator (NS, CT, or KS). Interobserver reliability for the eight-subtype classification was evaluated using Cohen's kappa (*κ*), calculated from independent first-pass assignments before consensus. For continuous morphometrics (ACP length, base width), ICC was calculated using a single-measure, two-way mixed-effects model with absolute agreement. All data were anonymized prior to analysis.

### 2.7 Statistical analysis

Statistical analyses were performed using the Stata/BE 17.0 (StataCorp LLC, College Station, TX, USA). Continuous variables are expressed as mean ± standard deviation (SD) and compared using independent t-tests. Categorical data were analyzed using *χ*^2^ or Fisher's exact test, as appropriate. Statistical significance was set at a two-tailed *p*-value <0.05.

## 3 Results

Two hundred Thai patients (400 ACPs) were included in this study. The cohort had an equal distribution of sexes, with 100 males and 100 females, and a mean age of 31.02 ± 21.97 years (range: 10–102 years). The morphometric characteristics of the ACP differed significantly according to sex. Male patients demonstrated significantly larger ACP dimensions, including both base width (9.09 ± 1.61 mm vs. 8.54 ± 1.39 mm, *p* = 0.015) and length (13.23 ± 1.72 mm vs. 12.61 ± 1.64 mm, *p* = 0.010), compared to female patients (Table 2).

**Table 2:** Demographic and morphometric characteristics of the study population.

Characteristics	Total	Male	Female	*p*-value
Number of patients (ACP sides)	200 (400)	100 (200)	100 (200)	1.000
Age, years (mean ± SD, range)	31.02 ± 21.97 (10—102)	30.88 ± 21.38 (10—93)	31.16 ± 22.65 (10—102)	0.929
ACP base width, mm (mean ± SD)	8.82 ± 1.52	9.09 ± 1.61	8.54 ± 1.39	0.015
ACP length, mm (mean ± SD)	12.92 ± 1.70	13.23 ± 1.72	12.61 ± 1.64	0.010
Presence of ACP pneumatization, *n* (%)				
– Total	124 (30.8%)	61 (30.5%)	62 (31%)	1.000
– Bilateral*	10 (5.0%)	5 (2.5%)	5 (2.5%)	1.000
Presence of calcification, *n* (%)				
– Middle clinoid process	23 (5.8%)	12 (6%)	11 (5.5%)	1.000
– Interclinoid calcification	35 (8.8%)	18 (9%)	17 (8.5%)	1.000

* Percentages of bilateral ACP cases were calculated based on the total number of patients (*n* = 200), whereas other values were calculated based on the number of ACPs. ACP, anterior clinoid process; SD, standard deviation.

ACP pneumatization was identified in 123 of the 400 ACPs (30.75%). The prevalence of pneumatization was nearly identical between the sexes (30.5% in males and 31.0% in females, *p* = 1.000), and bilateral pneumatization was present in 10 patients (5% of the cohort), evenly split between male and female individuals. Calcified middle clinoid processes and interclinoid calcifications were noted in 5.8% and 8.8% of ACPs, respectively, with no statistically significant sex differences (*p* = 1.000).

**Figure 3:**
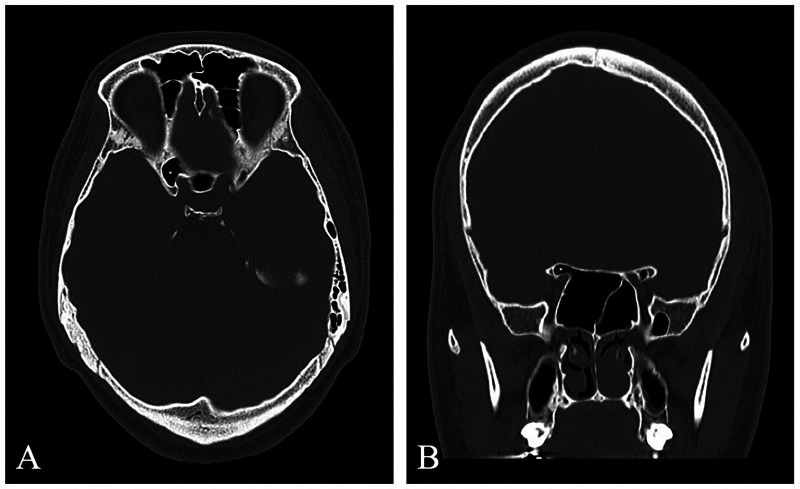
Bilateral type 2 anterior clinoid process (ACP) pneumatization. **(A)** Axial CT image showing left ACP pneumatization via the optic strut with ≤50% involvement (Type 2a, arrow) and right ACP pneumatization via the optic strut with >50% involvement (Type 2b, asterisk). **(B)** Coronal CT image of the same patient confirming left Type 2a and right Type 2b ACP pneumatization.

Using the proposed eight-grade classification system for ACP pneumatization, the most prevalent subtype was Type 0, indicating the absence of pneumatization, which was observed in 277 ACPs (69.25%). Type 1, representing pneumatization limited to the optic strut without extension into the ACP body, was the second most common type and was found in 64 ACPs (16%) (Figure 1). Subtypes indicating partial pneumatization of the ACP body via the optic strut were categorized as Type 2a (≤50% ACP involvement), seen in 24 ACPs (6%), and Type 2b (>50% involvement), found in 20 ACPs (5%) (Figure 3).

**Figure 4:**
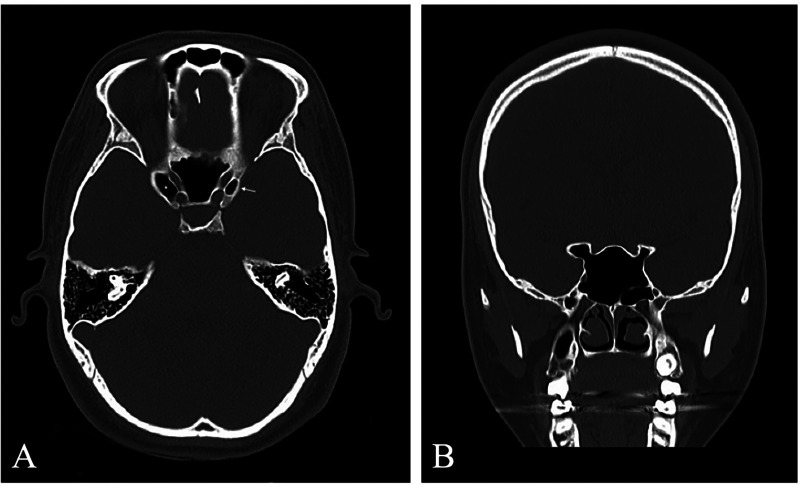
Type 3b anterior clinoid process (ACP) pneumatization **(A)** axial CT image demonstrating right ACP pneumatization through the planum sphenoidale with >50% ACP involvement (type 3b, asterisk). **(B)** Coronal CT image showing pneumatization extending from the sphenoid sinus through the planum sphenoidale into the ACP (Type 3b, asterisk).

**Figure 5:**
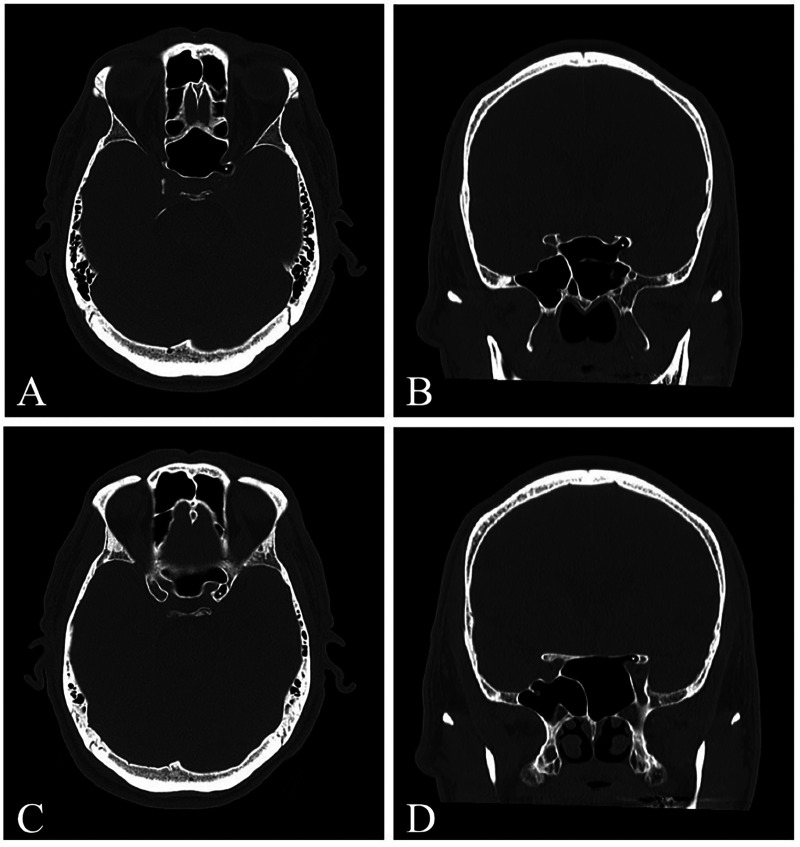
Type 4b anterior clinoid process (ACP) pneumatization **(A,B)** axial and coronal CT images demonstrating pneumatization through the optic strut (asterisks). **(C,D)** Axial and coronal CT images of the same patient demonstrating pneumatization through the planum sphenoidale (asterisks). Together, these findings represent combined optic strut and planum pneumatization with >50% ACP involvement (Type 4b).

Pneumatization patterns extending through the planum sphenoid were less common. These included Type 3a (≤50% ACP involvement via the planum), seen in three ACPs (0.75%), and Type 3b (>50% involvement), found in five ACPs (1.25%) (Figure 4). Complex pneumatization involving both the optic strut and planum sphenoidale was rare, comprising Type 4a (≤50% combined involvement, 2 ACPs, 0.5%) and Type 4b (>50% combined involvement, 5 ACPs, 1.25%) (Figure 5, Table 3).

**Table 3:** Distribution of anterior clinoid process (ACPs) pneumatization by proposed classification (*n* = 400 ACPs).

Pneumatization type	Male, n		Female, n		Total, n (%)
	Right	Left	Right	Left	
0	70	69	71	67	277 (69.25%)
1	17	16	13	18	64 (16%)
2a	2	4	8	10	24 (6%)
2b	6	6	5	3	20 (5%)
3a	1	1	1	0	3 (0.75%)
3b	4	1	0	0	5 (1.25%)
4a	0	0	1	1	2 (0.5%)
4b	0	3	1	1	5 (1.25%)
Total, n	100	100	100	100	400 (100%)

No statistically significant differences in the pneumatization subtype distribution were observed between the left and right sides or between sexes. Among the 124 pneumatized ACPs, most were unilateral. Although the prevalence of optic strut-related pneumatization (Types 1, 2a, and 2b) was higher than that of planum-related or combined types, the latter still accounted for a clinically relevant minority of cases.

The reliability analysis demonstrated substantial agreement between the two independent observers for the proposed eight-subtype classification system, with an overall Cohen's Kappa of 0.733 (95% CI: 0.69–0.78). When analyzed by specific subtypes, agreement was highest for Type 4b (*κ* = 0.888; 95% CI: 0.67–1.00) and Type 0 (*κ* = 0.852; 95% CI: 0.79–0.91). As expected, complex or smaller pneumatization patterns, such as Type 2a, showed moderate agreement (*κ* = 0.469; 95% CI: 0.28–0.66). Regarding morphometric measurements, interobserver reliability was good. The ICC for ACP length was 0.780 (95% CI: 0.71–0.83) for the right side and 0.756 (95% CI: 0.68–0.81) for the left side. Similarly, the ACP base width showed consistent reproducibility, with ICC values of 0.704 (95% CI: 0.62–0.77) for the right side and 0.690 (95% CI: 0.60–0.76) for the left side. Detailed reliability metrics are presented in Supplementary Table 1.

## 4 Discussion

In this cohort of Thai patients aged ≥10 years, ACP pneumatization was identified in 30.8% of 400 ACPs**,** with bilateral involvement in 5% of patients. This prevalence is at the higher end of a previously reported series. Importantly, all eight subtypes were represented, underscoring the ability of this refined system to capture the full spectrum of pneumatization patterns. Most pneumatization originated through the optic strut route (types 1, 2a–b, and 4a–b), comprising 28.8%, whereas pneumatization involving the planum sphenoidale route (types 3a–b and 4a–b) was relatively uncommon (3.8%). Morphometric analysis revealed sex-related differences, with males exhibiting greater ACP length and base width; however, no significant sex differences were observed in the prevalence of the middle clinoid process or interclinoid calcification.

Prevalence estimates of ACP pneumatization are theoretically more detectable in imaging-based studies, particularly CT scans, which provide a high-resolution evaluation of the skull base anatomy compared to cadaveric dissections. Age and ethnicity appear to influence prevalence, whereas sex differences remain uncertain.

Developmentally, pneumatization is consistently absent in children aged <10 years, as demonstrated in a pediatric CT study by Rennert et al. (7). Infants have indicated that small sphenoidal air spaces may be present early; however, clinically meaningful ACP pneumatization emerges only after late childhood (3, 7). Consequently, studies that include children have often reported an artificially low prevalence. In terms of ethnic variation, the prevalence in our Thai cohort (30.8%) is similar to the Brazilian data [25.5% reported by da Costa et al. (3)] and Turkish data [24% reported by Düz et al. (10)], but higher than that in the Japanese series [9.2% reported by Mikami et al. (6)]. These differences likely reflect both the true population variability and differences in classification sensitivity. With respect to demographic and laterality factors, these results demonstrated that neither sex nor side significantly influenced the prevalence or subtype of ACP pneumatization. This observation is consistent with previous studies, which likewise found no consistent differences between males and females or between the right and left sides (5, 6, 9).

Several classification systems of ACP pneumatization have been proposed, each with distinct strengths and limitations. Mikami et al. (6) first introduced a route-based classification that described pneumatization through the optic strut, planum sphenoidale, or both, and emphasized its potential origin from either the sphenoid or ethmoid sinuses. However, this system did not recognize optic strut–only pneumatization as a separate entity, which contributed to the relatively low reported prevalence of 9.2% in the Japanese cohort. Abuzayed et al. (5) later proposed a volume-based system ranging from Type 0 to Type III, with an overall prevalence of 9.6%. Although useful for describing the extent of pneumatization, this system does not differentiate whether the optic strut is involved.

More recently, da Costa et al. (3) developed a comprehensive classification that combined both the route and the degree of pneumatization. In their large cohort of 597 patients (1194 ACPs), the overall prevalence was 25.5%, which is consistent with the 30.8% prevalence in our present series. Their classification included Type 1 (optic strut only), Type 2A (≤50% ACP involvement via the optic strut), Type 2B (>50% via the optic strut), and Type 3 (planum route with or without optic strut involvement) (14). These findings show a similar distribution, with the prevalence of types 1 and 2 (21%) approximating da Costa's combined rates of Types 1 and 2A/B (18%), whereas our types 3a–b and 4a–b (3.8%) correspond to da Costa's Type 3 (3.1%). Importantly, Chaddad-Neto F. et al. (11) identified poor inter-observer reproducibility for Types 2A and 3, which was confirmed in a subsequent reproducibility study, where the Fleiss kappa values were the lowest for Types 2A (0.35) and 3 (0.39). By explicitly separating planum-only pneumatization (3a/3b) from combined planum and optic strut pneumatization (4a/4b), the refined classification may facilitate more standardized reporting and potentially improve interobserver agreement, particularly for planum-related patterns. From a practical standpoint, distinguishing planum-only vs. combined-route pneumatization may inform preoperative communication and operative preparedness, because combined-route patterns may imply a broader potential interface for sinonasal communication during drilling, while greater longitudinal extent (>50%) may warrant increased awareness regarding reconstruction preparedness. A side-by-side comparison of existing systems and our proposed classification is summarized in Table 4, which highlights the added resolution of the grading scheme to distinguish the route and degree of involvement. Nonetheless, as with all radiological classifications, neither da Costa's nor the present study could reliably distinguish whether the pneumatization originated from the sphenoid sinus or the ethmoid sinus, which remains a study limitation.

**Table 4:** Comparative classification systems for anterior clinoid process pneumatization.

Mikami et al. (6)	Abuzayed et al. (5)*	Da Costa et al. (3)	Present classification
0	0	0	0
- (Separate OS pneumatization from ACP pneumatization)	- (Not mentioned)	1	1
I	I	2A	2a
	II-III	2B	2b
II	I	3	3a
	II-III		3b
III	I		4a
	II-III		4b

* Abuzayed types reflect degree only and do not encode route. ACP, anterior clinoid process; OS, optic strut.

Anterior clinoidectomy is a technically demanding operation that requires precise anatomical knowledge of the ACP and its complex relationship with surrounding structures (15–17). Pneumatization of the ACP adds further complexity since removal of a pneumatized clinoid may create direct communication between the subarachnoid space and the sphenoid sinus, significantly increasing the risk of CSF leakage (9, 18). Various strategies have been proposed for mitigating this risk. Da Costa et al. (3) advocated stepwise clinoidectomy with meticulous reconstruction, particularly when pneumatization was present, and highlighted that reconstruction should be tailored to the pneumatization route. Chi et al. introduced the “yo-yo” technique, in which a temporalis muscle plug is anchored and retracted into a pneumatized optic strut to seal the communication (19). These findings support the potential value of preoperative classification for anticipating optic strut or planum communication and informing surgical planning (e.g., preparedness for reconstruction), although clinical impact was not assessed in this radiologic study.

An important insight from this analysis is that ACP pneumatization may influence the angle and degree of optic canal exposure when assessed from an intrasphenoid perspective. Endoscopic endonasal drilling has traditionally been guided by the overall pneumatization of the sphenoid sinus, categorized as conchal, presellar, or sellar types (20), and by the configuration of the intersphenoid septa (21). These findings suggest that ACP pneumatization should be considered when planning optic canal decompression. According to our classification, the extent of pneumatization correlates with the degree of circumferential optic canal exposure (14): in Types 0 and 1, only the inferior wall is accessible; in Type 2, the latero-superior wall is additionally exposed via the lateral opticocarotid recess, corresponding to optic strut pneumatization; in Type 3, the medio-superior wall becomes approachable through the superior opticocarotid recess, representing ACP pneumatization extending from the planum; and in Type 4, nearly the entire circumference of the optic canal can be visualized. Integrating the ACP pneumatization subtype with optic canal accessibility may therefore serve as a predictive tool for endoscopic surgeons, providing valuable guidance for preoperative planning in cases requiring optic canal decompression and potentially enhancing surgical freedom during the endonasal approach (22).

This study has a few limitations. First, this was a retrospective analysis conducted at a single center in Thailand, which may have limited the generalizability of the findings to other populations. Second, intraoperative validation was not performed; therefore, individual pneumatization subtypes could not be directly correlated with intraoperative findings or postoperative complication such as CSF leakage or the degree of optic canal exposure. Third, the low prevalence of subtypes 3 and 4 (<1.5%) limits statistical inferences for these specific variants; therefore, although the system covers the full radiological spectrum, its reproducibility requires confirmation in larger, multi-ethnic cohorts. Fourth, the series excluded pediatric patients, which is consistent with prior evidence that ACP pneumatization is developmentally absent before this age. However, this limits the findings to adolescents and adults. Finally, sphenoid sinus subtypes or intersphenoid septal variations were not analyzed, nor could we definitively differentiate pneumatization of the sphenoid vs. posterior ethmoid (Onodi) origin, which may occasionally be misclassified on CT because of thin bony partitions and potential false negatives.

## 5 Conclusion

Preoperative recognition of ACP pneumatization alerts the surgeon to potential risks, such as CSF leakage following clinoidectomy, and may be leveraged to enhance surgical access, particularly around the optic canal. In this regard, our proposed eight-subtype classification provides a more detailed framework for describing ACP pneumatization routes and their extent. ACP pneumatization is a relatively common anatomical variation with a comparable prevalence between males and females in our series. Although ACP pneumatization's surgical implications are evident for both transcranial and endoscopic endonasal approaches, the actual effect size on postoperative complications and surgical accessibility remains unclear, and concordance with intraoperative findings was not assessed in this radiologic study and warrants further investigation through clinical correlations.

## Data Availability

The raw data supporting the conclusions of this article will be made available by the authors, without undue reservation.
