# Supplementary material for: A refined radiological classification of anterior clinoid process pneumatization
Source: Front Surg. 2026 Apr 13;13:1726314. doi: 10.3389/fsurg.2026.1726314 (PMC13111364; doi:10.3389/fsurg.2026.1726314)
Supplement: Supplementary file 1 [file Table1.docx]

**Supplementary Table 1:** Interobserver reliability statistics for the proposed ACP pneumatization classification and morphometric measurements.

| **Classification Variable** | **Cohen’s Kappa (κ)** | **95% CI** |
| --- | --- | --- |
| **Overall (8 Subtypes)** | **0.733** | **0.69 – 0.78** |
| Type 0 | 0.852 | 0.79 – 0.91 |
| Type 1 | 0.668 | 0.55 – 0.79 |
| Type 2a | 0.469 | 0.28 – 0.66 |
| Type 2b | 0.704 | 0.49 – 0.92 |
| Type 3a | 0.663 | 0.17 – 1.00 |
| Type 3b | 0.568 | 0.14 – 1.00 |
| Type 4a | 0.799 | 0.41 – 1.00 |
| Type 4b | 0.888 | 0.67 – 1.00 |
|  |  |  |
| **Morphometric Variable** | **ICC** | **95% CI** |
| **ACP Length** |  |  |
| Right Side | 0.780 | 0.71 – 0.83 |
| Left Side | 0.756 | 0.68 – 0.81 |
| **ACP Base Width** |  |  |
| Right Side | 0.704 | 0.62 – 0.77 |
| Left Side | 0.690 | 0.60 – 0.76 |
| ACP, anterior clinoid process; CI, confidence interval; ICC, intraclass correlation coefficient. | | |
